# A scoping review of the social determinants of maternal health in the MENA region

**DOI:** 10.11604/pamj.2024.47.205.42499

**Published:** 2024-04-23

**Authors:** Chaimae Moujahid, Jack Edward Turman, Loubna Amahdar

**Affiliations:** 1 Laboratory of Health Sciences and Technologies, Higher Institute of Health Sciences, Hassan First University of Settat, Settat, Morocco,; 2 Department of Social and Behavioral Sciences, Richard M Fairbanks School of Public Health, Indiana University, Indianapolis, United States of America,; 3 Department of Pediatrics, School of Medicine, Indiana University, Indianapolis, United States of America

**Keywords:** Maternal health, iniquity, genders, violence, health care system

## Abstract

One crucial step to improving maternal health outcomes in any region is understanding the social determinants of maternal health, which vary significantly across the world´s geographical areas and within individual countries. The variability in these determinants is manifested in the Middle East and North Africa (MENA) region. Using a scoping review process, we identified articles analyzing social factors influencing maternal health outcomes in the MENA region. A total of 50 articles were included in this review. Several social factors impact independently or in association with maternal health outcomes or utilization of maternal health in the MENA region. These factors include: residing in an area of conflict, residing in a rural region, low accessibility and quality of health care, low level of education, antagonistic relationship with spouse and family-in-law, cultural practices such as female genital mutilation and early marriage, traditional practices, and beliefs, low household wealth, women´s financial security, women's bad childbirth history, and interpersonal violence. Multi-sector collaboration across governmental ministries, non-governmental organizations, local authorities, healthcare delivery programs, and community members is critical to creating long-term solutions in maternal health for MENA nations. Together they must address traditional practices harmful to women, poor accessibility, availability, and affordability of health services. To benefit women, a long-term commitment of organizations at local, national, and international levels to social investments in women´s education, financial status, and cultural norms is recommended for MENA nations.

## Introduction

The MENA region has seen a 50% decrease in maternal and under-five mortality rates between 1990 and 2015, but ongoing wars and disease outbreaks have exacerbated public health issues, particularly for women and children. These crises have also impacted the implementation of children's rights to health, despite the global decrease in maternal mortality rates [1]. In Mena region, Wealthier countries are doing better than poorer countries. For example, the United Arab Emirates reports in 2017 maternal mortality ratio of 3/100,000 live births while Yemen reports an MMR of 164/100,000 live births. A critical step to improving maternal health outcomes in any region is understanding the social determinants of maternal health. Understanding these determinants allows for developing and implementing intervention strategies to address social systems influencing maternal health. When women are valued, enabled, and empowered in all domains, gender equality and sustainable health development can be achieved [2]. Our review helps identify unique social determinants of maternal health within nations and common determinants across the region, all of which must be addressed to improve regional maternal health outcomes. Our findings can help government and non-government organizations implement regional health and social programs that address social inequalities such as the distribution of power, income, goods, and services, as defined by Marmot [3].

## Methods

**Study design**: in this study, a scoping review was selected to map existing literature on the social determinants of maternal health in the Middle East and North Africa region [4]. The first reason for scoping is to explore the research activity's extended range and nature, while the second is to determine whether a systematic review is feasible and of value. The third and fourth reasons for exploratory analysis are seen as distinct and intrinsically valuable objectives. The third reason requires meticulous selection of studies directly relevant to the research questions posed, ensuring exhaustive coverage of specific areas of interest. On the other hand, the fourth reason focuses on data representation, facilitating the synthesis of information and the identification of gaps in the literature to guide future research directions [5,4]. For our study, Arksey and O'Malley's framework for conducting a scoping review was used to identify knowledge gaps in understanding the social determinants of maternal health in the MENA region [6]. Furthermore, the PRISMA extension for scoping reviews (PRISMA-ScR) were used to present our results [7].

**Figure 1:**
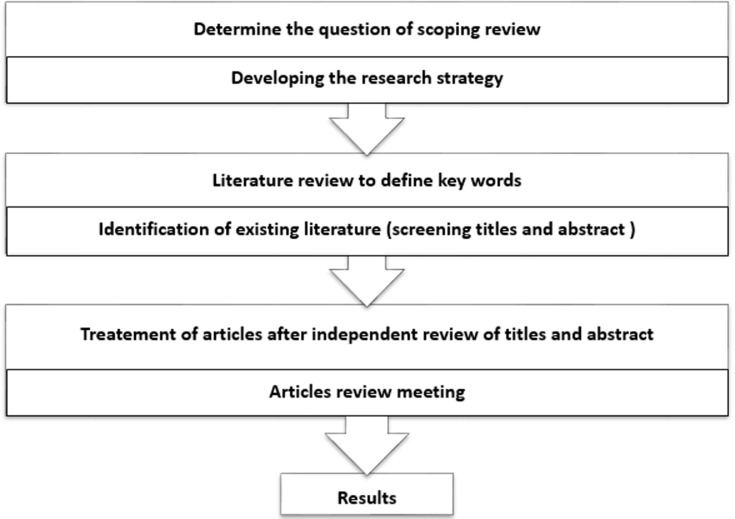
steps in operating the scoping review on social determinants of maternal health in Middle East and North Africa

**Literature research**: our search strategy was conducted using the following databases to identify peer-reviewed published papers: Springer, web of Science, PubMed, Scopus, J. Store, and Google Scholar. References in all the relevant identified studies were hand-searched to identify additional studies. We also searched for international surveys with data on social determinants of maternal health from any MENA country using databases of UNICEF, Demographic and Health Surveys (DHS), and the World Bank health statistics. Searches included combinations of relevant keywords: social determinants, maternal health, cultural factors, Middle East, North Africa, maternal status, maternal health in variety with the names of all WHO Eastern Mediterranean Region and World Bank MENA regions. Search words were combined using Boolean operators (AND, NOT, OR) to refine the results [8] (Figure 1).

**Inclusion and exclusion criteria**: the following inclusion criteria were used: articles and reports on maternal health in MENA, articles discussing social determinants of maternal health, reports written in English, and quantitative and qualitative studies. According to the following criteria, articles and reports with the main focus other than maternal health in MENA and articles not in English were excluded.

**Figure 2:**
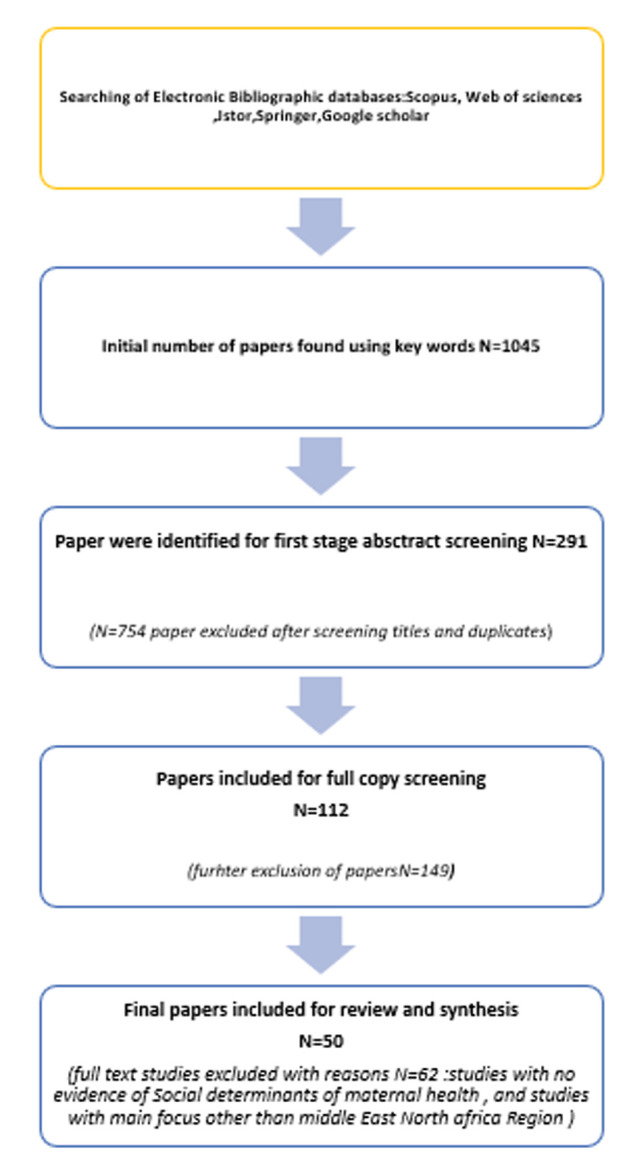
flowchart of the number of literature searched and selected

**Study selection**: the initial search of databases revealed 1045 pieces of literature, 903 of which were excluded after screening their titles and abstracts. Complete copies of 112 pieces of kinds of literature were read first independently and then reviewed again by the authors, which resulted in the further exclusion of 62 papers as they did not meet our inclusion criteria. The final papers included in this review were narrowed down to 50 total pieces (Figure 2).

**Figure 3:**
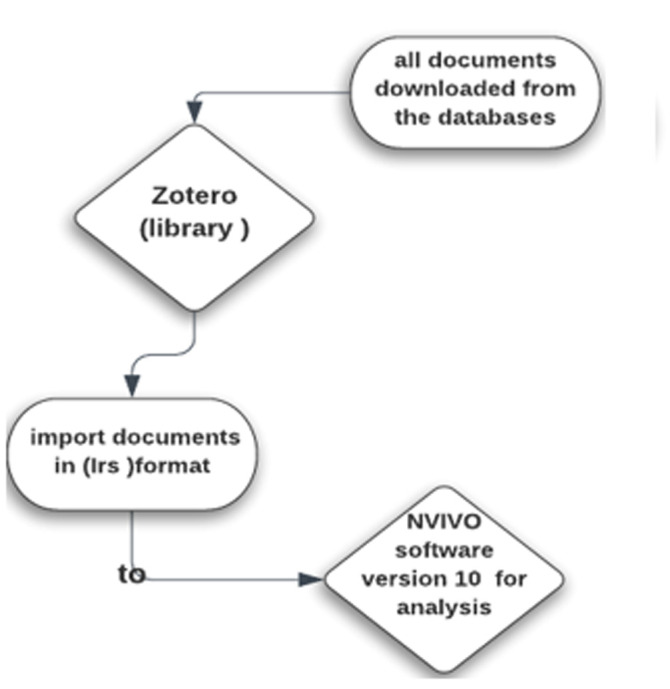
the steps of management and analysis of our database

**Management and analysis**: after deleting duplicates, retrieved sources were imported into Zotero. Titles and abstracts were screened to ensure they pertained to MENA countries and presented evidence about social determinants of maternal health. After providing that the data research met the inclusion criteria, the articles were transported from Zotero (IRIS format) to NVIVO (version 10) software for qualitative analysis (Figure 3).

**Data extraction**: the following information was entered for each study: i) article identification (title, authors, years of data collection and publication, journal name, country); ii) respondents, research design, setting, sample size, study population, and social determinants of maternal health (community context, culture, social values, individual attributes, family characteristics, health services, structural determinants).

## Results

**Figure 4:**
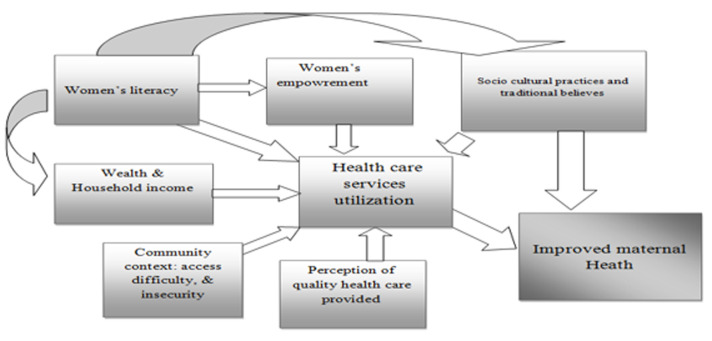
pathways through which determinants influence maternal health

The articles reviewed in this study included: systematic reviews, cross-sectional studies, case-controlled studies, randomized controlled trials, non-randomized cluster intervention studies, narrative reviews, and qualitative and methodological studies (Table 1, Table 1.1, Table 1.2, Table 1.3, Table 1.4, Table 2). An iterative procedure was used for data extraction. After reading every paper that qualified for inclusion and classifying them into general categories according to the primary data type (social determinants of maternal health), one author (CM) extracted pertinent text from the article sections that offered suggestions, techniques, or strategies for enhancing the quality of the data. After that, at least one more author (CM, JT, or LA) went over each item to make sure the extraction was finished and accurate before coding, as well as to guarantee consistency and quality. Discussion was used to determine any differences in the data extraction process. One author (CM) conducted a thematic analysis to find themes, which were then narratively reported and verified by two other authors (JT and LA). The most critical social determinants identified in this research included “health care services,” “individual attributes and health care behavior,” “education and wealth,” employment and income,” cultural practices and traditional beliefs,” and “violence and poverty” (Figure 4).

**Table 1:** characteristics of included studies

Author	Year	Title	Country	Social determinants
Hijazi *et al*.	2018	Determinants of antenatal care attendance among women residing in highly disadvantaged communities in Northern Jordan: a cross-sectional study	Jordan	-Health care services utilization -Education
Kabakian Khasholian *et al*.	2005	A simple way to increase service use: triggers of women’s uptake of postpartum services	Lebanon	-Health care services -Maternal health knowledge -Education
Gopalan *et al*.	2019	Associations between acute conflict and maternal care usage in Egypt: an uncontrolled before-and-after study using demographic and health survey data.	Egypt	- Individual attributes - Health care (ANC services usage, institutional postnatal care)
Ghazal-Aswad *et al*.	2011	Confidential enquiries into maternal mortality in the United Arab Emirates: a feasibility study.	United Arab Emirates	- Maternal health outcomes
Wilunda *et al*.	2017	Barriers to utilisation of antenatal care services in South Sudan: a qualitative study in Rumbek north county.	South Sudan	-Healthcare services (Access to ANC) -Income -Transportation -Environmental (infrastructure) -Maternal health knowledge
Celik *et al*.	2000	The socio-economic determinants of maternal health care utilization in Turkey.	Turkey	-Income -Household Wealth -Individual attributes (parity, Ethnicity) -Education -Health care -Environmental (geographic accessibility)
Zalv and *et al*.	2019	determinants and causes of maternal mortality in Iran based on ICD-mm: a systematic review.	Iran	-Health care services -Maternal health outcomes (Maternal health complications, Maternal deaths …) -Income
Bruno *et al*.	2019	Determinants of health facility utilization at birth in South Sudan	South Sudan	-Health care services (ANC, SBA) -Education -Income -Individual attributes (parity)
Hussein *et al*.	2018	Women’s experiences of childbirth in Middle Eastern countries: a narrative review	Middle Eastern Countries	-Health care services -Maternal health knowledge

**Table 1.1:** characteristics of included studies

Author	Year	Title	Country	Social determines
Mugo *et al*.	2016	Factors associated with different types of birth attendants for home deliveries	South-Sudan	-Health care services (Accessibility, quality of care) -Income -Education -Maternal heath knowledge
Hagiwara *et al*.	2013	Is the Maternal and Child Health (MCH) handbook effective in improving health-related behavior? Evidence from Palestine.	Palestine	-Health care services (Accessibility and utilization) -Maternal health knowledge -Education
Gopalan *et al*.	2017	Maternal and neonatal service usage and determinants in fragile and conflict-affected situations: a systematic review of Asia and the Middle-East.	Middle East (Iraq – Yemen, and Palestine)	-Political context (Instability) -Health care services (Accessibility) -Income -Transportation -Autonomy -Maternal health knowledge
Khadr *et al*.	2009	Monitoring socioeconomic inequity in maternal health indicators in Egypt:	Egypt	-Health care -Income
Green *et al*.	2007	change and continuity: childbirth and parenting across three generations of women in the United Arab Emirates	United Arab Emirates	-Individual attributes (maternal age at first birth …) -Health care -Education
Bottcher *et al*.	2018	Maternal mortality in the Gaza strip: a look at causes and solutions	Gaza	-Health care services (quality of health care, ANC accessibility…) -Individual attributes ( parity, age )
Dejong *et al*.	2012	Effects of reproductive morbidity on women’s lives and costs of accessing treatment in Yemen. reprod.	Yemen	-Health care services -Income
Awadalla H *et al*.	2009	Evaluation of maternal and child health services in El-Minia City, Egypt	Egypt	-Health care services -Income
El Sheikh *et al*.	2015	Factors influencing the utilization of maternal health care services by Nomads in Sudan	Sudan	-Health care system -Poverty -Income -Sociocultural factors -Education -Maternal Health knowledge -Gender norms -Residence and geographic accessibility

**Table 1.2:** characteristics of included studies

Author	Year	Title	Country	Social determines
Renaudin *et al*.	2007	Ensuring financial access to emergency obstetric care: three years of experience with obstetric risk insurance in Nouakchott, Mauritania	Mauritania	-Health care system
Zaouaq *et al*.	2017	Women and access to reproductive health care in Morocco.	Morocco	-Health care system -Cultural factors -Education -Income
El Hamdani *et al*.	2013	Prenatal care in the city of Marrakech.	Morocco	-Health care system -Sociocultural factors -Income -Residence
Mugo *et al*.	2015	Prevalence and risk factors for non-use of antenatal care visits: analysis of the 2010 South Sudan household survey	South Sudan	-Health care system -Individual attributes -Residence -Health knowledge -Education
Giacaman *et al*.	2008	Palestinian women’s pregnancy intentions: analysis and critique of the demographic and health survey 2004	Palestine	-Individual attributes -Health behavior
Mustafa *et al*.	2015	Factors associated with antenatal and delivery care in Sudan: analysis of the 2010 Sudan household survey	Sudan	-Health care system -Education -Income and wealth -Individual attributes
Shabila *et al*.	2014	Women’s views and experiences of antenatal care in Iraq: a q methodology study	Iraq	-Health care system (availability, accessibility, ANC) -Maternal Health knowledge
Abdulrahim *et al*.	2019	Regional inequalities in maternal and neonatal health services in Iraq and Syria from 2000 to 2011.	Iraq	-Health care system -Education -Income
Douki *et al*.	2007	Women’s mental health in the Muslim world: cultural, religious, and social issues	Arab countries Tunisia and United Arab Emirates (UAE)	-Maternal health outcomes -Sociocultural factors -Education
Balinska *et al*.	2019	Reproductive health in humanitarian settings in Lebanon and Iraq: results from four cross-sectional studies, 2014-2015.	Iraq and Lebanon	-Health care system -Education -Socio-economic factors
Livani *et al*.	2009	The status and progress of women in the Middle East and North Africa;	MENA region	-Income -Gender Norms -Transportation -Residence

**Table 1.3:** characteristics of included studies

Author	Year	Title	Country	Social determine
Mohammad *et al*.	2014	Factors associated with birth weight inequalities in Jordan	Jordan	-Education -Individual attributes -Income
Islam *et al*.	2014	Advanced maternal age and risks for adverse pregnancy outcomes: a population-based study in Oman	Oman	-Individual attributes -Maternal health outcomes
Dinçer *et al*.	2014	Women’s education: harbinger of another spring? evidence from a natural experiment in Turkey	Turkey	-Education -Health care Access -Maternal health knowledge
Tajik *et al*.	2012	Inequality in maternal mortality in Iran: an ecologic study	Iran	-Income -Education -Maternal outcomes
Alosaimi *et al*.	2016	Measures of maternal socioeconomic status in Yemen and association with maternal and child health outcomes	Yemen	-Maternal health outcomes -Education -Incomes -Residence -Living conditions
Chaaya M *et al*.	2002	Postpartum depression: prevalence and determinants in Lebanon	Lebanon	-Education -Income -Maternal health outcomes
Cresswell J *et al*.	2015	Trends in health facility deliveries and caesarean sections by wealth quintile in Morocco between 1987 and 2012	Morocco	-Health care utilization -Wealth -Income
Khawaja M *et al*.	2007	Types of cultural capital and self-rated health among disadvantaged women in outer beirut, Lebanon	Lebanon	-Women health outcomes -Individual attributes -Social context
Buvinic S *et al*.	1997	Female-headed households and female-maintained families: are they worth targeting to reduce poverty in developing Countries?	Developing countries (including MENA region)	-Women empowerment (Women headed households (WHH)) -Poverty -Income
Rashad A *et al*.	2017	Socioeconomic inequalities in maternity care utilization: evidence from Egypt, Jordan and Yemen	Egypt, Jordan and Yemen.	-Maternal health outcomes -Health care system -Poverty -Education -Income -Residence -Socioeconomic factors
Gee S *et al*.	2018	We need good nutrition but we have no money to buy food”: sociocultural context, care experiences, and newborn health in two UNHCR-supported camps in South Sudan	South Sudan	-Sociocultural factors -Maternal health outcomes -Health care system (accessibility, availability, quality of care) -Environmental and contextual factors: poor nutrition)

**Table 1.4:** characteristics of included studies

Author	Year	Title	Country	Social determine
Yasin *et al*.	2013	Female genital mutilation among Iraqi Kurdish women: a cross-sectional study from Erbil City	Iraq	-Cultural and traditional practices -Health outcomes -Household Education level -Maternal health knowledge
Satti A *et al*.	2006	Prevalence and determinants of the practice of genital mutilation of girls in Khartoum, Sudan.	Sudan	-Socioeconomic factors -Education -Sociocultural practices -Health outcomes -Social context
Al Shahethi A *et al*.	2019	Maternal, prenatal and traditional practice factors associated with perinatal mortality in Yemen.	Yemen	-Sociocultural practices -Individual attributes (maternal age, parity,) -Living conditions -Health outcomes
Serizawa A *et al*.	2014	cultural perceptions and health behaviors related to safe motherhood among village women in eastern Sudan: ethnographic study.	Eastern Sudan	-Socio economic factors -Sociocultural and religious factors -Maternal health behaviors -Health care system (utilization)
Ertem M *et al*.	2008	the factors associated with adolescent marriages and outcomes of adolescent pregnancies in Mardin Turkey	Turkey	-Sociocultural practices (Early Marriage,) -Education
Samari *et al*.	2018	Longitudinal determinants of married women’s autonomy in Egypt	Egypt	-Women Autonomy -Household living conditions -Residence -Household Wealth
Chiang *et al*.	2012	Improvements in the status of women and increased use of maternal health services in rural Egypt.	Egypt	-Social context (family support) -Women’s empowerment (Women’s family status) -Health care system (Accessibility and utilization) -Residence -Education -Individual attributes -Gender norms (conjugal violence)
Esen Danaci *et al*.	2002	Postnatal depression in Turkey: epidemiological and cultural aspects	Turkey	-Psychological Maternal health -Living conditions -Women family relationship (spouse and family in law) -Individual attributes (women health stories, immigration)
Çivic S *et al*.	2008	The Frequency of violence against women and the factors affecting this: a study on women who applied to two primary health care centers	Turkey	-Gender norms Violence -Individual attributes age, -Income -Education (ex : Husband education level ) -Family size
Hamzeh *et al*.	2008	Opinions of married women about potential causes and triggers of intimate partner violence against women. a cross-sectional investigation in an Iranian city	Iran	-Violence -Individual attributes -Health care system -Women’s employment -Education

**Table 2:** classification of number of studies by country

Study area	Number of articles
Jordan	02
Lebanon	02
Egypt	05
United Arab Emirates	02
Sudan	09
Turkey	05
Iran	03
Palestine	02
Yemen	04
Oman	01
Iraq	03
Morocco	03
Mauritania	01
More than one country of Mena region	08

**Health care system**: the lack of health care professionals, inaccessibility, and underutilization of services were the biggest problems for women in the MENA region. In Jordan, despite universal ANC coverage, geographic disparities are still a barrier. The utilization of ANC services was 2.37 times more likely among women living in the neighborhood served by maternal child health centers (MCH) [9]. Also, Lebanon has the lowest rate of mothers being discharged from hospital within 24 hours after giving birth and the low rate of postnatal consultation [10]. Furthermore, in the peri-conflict period in Egypt (2011-2012), a woman staying for a post-delivery period of at least 24 hours was 8% lower for peri-conflict births compared to pre-conflict births due to several factors caused by acute conflict [11]. Likewise, foreign women living in UAE contribute to the vast majority of maternal mortality (75%) compared to native-born citizens, and 60% of maternal deaths in the United Arab Emirates (UAE) occurred during the postpartum period [12]. South Sudan has one of the world's poorest health systems. Only 40.3% of pregnant women received ANC from a skilled provider [13]. Health insurance coverage contributes to the utilization of health care. In Turkey, having insurance coverage impacted the utilization of ANC positively and decreased the risk of giving birth at home [14]. In the MENA region, the percentage of skilled health personnel has increased from 72% to 80% to 89% in the past decade. However, disparities between MENA countries are well established [15]. Most of the articles reviewed show that women's home delivery and underutilizing SBA are the critical determinants of adverse maternal health outcomes. In Iran, for instance, SBA versus unskilled birth attendants were significant factors in association with maternal mortality [16]. Political instability has also discouraged mothers from utilizing services. Only a quarter (209/810) of the mothers had given birth at health facilities in South [17].

Fear of attacks, and geographical inaccessibility significantly impact maternal health care. Women cannot leave their children unattended at home, leading husbands to prevent giving birth in health facilities. This cycle of instability, fear, and displacement severely hampers women's ability to seek and receive necessary health services [13]. Women´s home delivery receives limited support from health professionals and the women experience anxiety and loneliness. Hospital policies have often prohibited women from having a companion of their choice during birth [18]. Throughout the analysis of a cross-sectional 2010 study in South Sudan. Thirty-six percent (36%) of deliveries were without assistance, 19% involved unskilled attendants, and 45% involved expert attendants. Factors such as mother's education, awareness of danger signs, and quality of prenatal care were associated with higher skilled birth attendance rates, emphasizing the importance of education and high-quality prenatal care [19]. In Palestine, maternal and child health services have significantly improved even in the face of political unrest and continued conflicts as of 2006. This includes a large number of newborns attended by qualified midwives, extensive prenatal care, and high rates of childhood vaccination. Considering the difficult conditions the area is facing, the achievements are especially significant [20]. According to a systematic review, Iraq (2011) showed that 70% of mothers did not seek any professional advice on newborn health [21].

However, there is a considerable decrease in MMR in MENA regions. In Egypt, deliveries assisted by skilled birth attendants increased by almost 70% from 42% in 1995 to 71% in 2005. This increasing trend was also accompanied by a decline in home delivery from more than 65% in 1995 to 34% in 2005 [22]. The coverage of Emirati women by qualified personnel at birth is universal across generations. Eleven-percent (11%) of the grandmothers reported giving birth in a hospital compared with 100% of daughters [23]. Women are affected by the inequality in health coverage contributes directly to the negative saturation of maternal health indicators in part of the MENA region. Unkind attitudes of health providers toward pregnant women in Palestine harm the utilization of health care services [20]. Distrust in physicians among families in Gaza might impact health indicators, including maternal mortality [24]. Yemeni women reportedly preferred public hospitals due to the presence of qualified staff. However, some women chose home delivery for feelings of security, family support, and lower costs [21]. Out-of-pocket contributions of households to health care costs are as high as 75% in Yemen, with the government contributing only 25% [25]. Costs and the real and perceived poor quality of care are significant barriers to ANC utilization in Sudan [13]. In El-Minia City, Egypt, the most reported cause of dissatisfaction with the services provided among clients of the urban center was the long waiting time. Despite higher utilization of the rural centers, histories of abortion and under-5-year moralities were more prevalent [26]. Affordability and care availability are critical barriers to accessing maternal care in MENA countries. In many countries of the MENA region, essential maternal care services are formally free of charge. However, formal out-of-pocket payments are required [27]. Mauritania's high cost of emergency obstetric care (EmOC) is a catastrophic health expenditure for households. 25% of the population survives on only $0.70 a day per household [28]. Likewise, disparities in the distribution of health resources existed inter-as in intra-countries. Morocco's bedding capacity is lower than other states in the Maghreb region, notably Tunisia and Algeria. Morocco's overall health care structures do not respond equitably to women's demands. The greater Casablanca region had the most significant number of gynecologists and midwives [29].

**Individual attributes and health care behavior**: maternal care could be available and affordable but sometimes not accessible due to various psychological and individual aspects, including maternal age, parity, health knowledge, previous obstetric history, and unwanted pregnancy. Women's awareness of complications and symptoms of pregnancy is higher among those who use health services than those who do not [30]. Maternal age (older), adverse history of pregnancy complications, poor perception of antenatal care at PHCCs (public primary health centers) all contributed in low utilization of health care services in Sudan [31,32], Palestine [33] and Iraq [34] respectively.

### Education and wealth

**Education level**: the lack of access to antenatal services is directly related to the proportion of women in the region with no formal education [35]. Women with even a few years of schooling have more self-confidence. They assume responsibility, communicate more with their husbands, and may have a higher status in the family [36]. Furthermore, educated women are more likely to assimilate messages about maternal health [20] and, therefore, are more likely to consult and monitor their pregnancy [30]. Women living in households headed by women with secondary education had more excellent antenatal care odds than those with no education [37]. Women's education has an impact not only on maternal health but also on -newborn health [23,38]. The highest number of maternal deaths occurred among illiterate women (Zalvand *et al*. 2019). In Sudan, unassisted delivery or delivery by an unskilled birth attendant was associated with mothers who had no schooling and who did not attend ANC during pregnancy [32]. Likewise, in Oman, 62% of the advanced-age mothers aged 35 and above had less than primary education related to an increased risk of adverse pregnancy [39]. Moreover, the education enrollment obligations of girls in Turkey have improved maternal and reproductive health issues [40]. An association was found between maternal death and the human development index (HDI), which refers to life expectancy, level of education, and income [41]. In Yemen, the risk of stillbirth, spontaneous abortion, infant mortality, and neonatal mortality decreased as women's educational levels increased [42]. In Lebanon, education has been shown to impact maternal outcomes. Less-educated women were more likely to have postpartum depression [43].

**Wealth/employment/poverty**: in MENA, the wealthiest 20 percent of mothers were almost 1.3 times more likely to have an SBA at delivery than mothers in the poorest 20% of the households (73.8% versus 95.3%) [15]. In Morocco, women from the wealthiest 20% of households were more than twice as likely to use facility-based delivery care and nearly three times more likely to deliver by cesarean section than those from the poorest 20% of households [44]. Even though maternal health in Egypt has improved, more educated affluent women had a higher rate of cesarean-sections and postpartum vitamin A supplements [22]. In South Sudan, the demand for payment for healthcare at some health facilities is one of the causes for the low utilization of health care services [13,32]. Furthermore, the impact of income on Lebanese women's health is well established. Likewise, women in the lowest quartile were significantly more likely to report poor health [45].

**Poverty**: poverty and its consequences on mother and child health are linked to women-headed households [46,47]. Yemeni women are paid the lowest in the rating of domestic workers' wages when compared to female workers from other countries. Yemeni domestic workers generally voice complaints about heavy workloads, inadequate or delayed salary payments, denial of legal rights, physical and psychological abuse, and isolation [48]. Women from economically better-off households are more likely to receive medical assistance at child delivery, but severity varied by country [49]. In Sudan, which is politically unstable due to civil war, women are forced to work both inside and outside the home to secure food and drink [50].

Cultural practices/traditional beliefs. at birth, many Muslim women are still exposed to discrimination because a girl cannot transmit the family name and is considered potentially dangerous for the honor of her family [36]. Female genital mutilation (FGM) is still widely practiced in Sudan, Egypt, Mauritania, and Iraq. Type I mutilation is the most common type practiced (99.6%) [51]. In Sudan, 66% of those who had undergone FGM [52] were classified as type III by the WHO [53]. Yemeni mothers who underwent FGM were almost three times more likely to experience perinatal death [54]. In Sudan, this practice leads to fistulae and birth complications that can result in maternal death or disabilities [27]. Despite the unhealthy consequences, FGM is strongly unmitigated [55].

Marriage at an early age, family size, culture, and gender inequity: the probability of having more than one stillbirth and child death was 5.6 and 3.66 times greater, respectively, among Turkish women who married at a young age compared to those who married later in life [56]. Early marriage is a predictor factor of low utilization of maternal health care services and causes high maternal death [27]. Women older than 18 at first marriage are more likely to make household decisions, have greater mobility, and have more financial autonomy [57]. Furthermore, many women in the MENA area must obtain permission from their husbands and in-laws to receive health treatment [58]. A bad relationship with a husband negatively impacts maternal health [59]. In Yemen, women who chewed khat during pregnancy had a higher risk of perinatal fatalities than those who did not. This societal practice increases the chance of fetal mortality [54].

**Violence**: in addition to the other determinants, girls and women are the primary victims of domestic violence [36]. In a study conducted in Konya, Turkey, eighty-four women out of 405 cases experienced violence. Abused women have poorer physical and mental health status than non-abused women [60]. Addiction of a partner, “unsatisfying sexual relationship,” and “husband's long-term unemployment” (92.2%,88.7%,83,00%, 75,9%) are respectively the proportion of women who “agreed” about the triggers of intimate partner violence against women IPVAW [61].

## Discussion

Maternal health conditions and their determinants are exaggerated in countries where conflict and war have conditioned community insecurity. These conditions restrict access to health services in Sudan, South Sudan, and Yemen. In addition, traditional practices and cultural beliefs aggravate the poor conditions for maternal health. In contrast, conditions in oil-producing countries have improved maternal health by universalizing the supply of care, increasing access to health services, and reducing socioeconomic disparities. These maternal health determinants are echoed by a large amount of literature worldwide [62]. In Pakistan, for example, adverse maternal health outcomes were related to women with poor spousal and family members relations and having a sizeable financial dependence on the husband and the family for access to health care facilities [63,64].

Furthermore, the husband´s absence from home (13%) and family hesitancy for referring women to health care services (16%) were factors contributing to maternal death [65]. Beyond the direct interpersonal relationships, it is found that the overall social environment is an important determinant that affects maternal health [66]. In addition to familial and social support, education is an essential determinant that affects maternal health behavior and is often related to socioeconomic status. As reported in this review, education represents an important determinant of maternal health in other developing countries. This was well established by several studies in Angola [67], Zambia [68], and Bangladesh [69]. Besides what was reported by the studies mentioned above, others concluded that religion should be integrated into the social determinants of the health framework [70]. Some of the political and other community interventions carried out by developing countries in the MENA region have improved access to health care services, which improves maternal health.

## Conclusion

Several important factors contribute to the maternal health improvements across the MENA region. Women's education is seen as a political priority, demonstrating the understanding of the critical role education plays in women's autonomy and the enhancement of maternal health outcomes. Last but not least, universal access to health care ensures that everyone, regardless of socioeconomic status, has access to the critical maternal health treatments. This all-encompassing strategy emphasizes that improving maternal health necessitates a multidimensional effort including not only the medical field but also a number of governmental and non-governmental organizations. Other sectors take proactive efforts to address their considerable effect on maternal health outcomes, rather than targeting the Ministry of Health exclusively. Using a collaborative approach, policies and programs that promote women's education, eliminate socioeconomic inequities, and guarantee universal access to healthcare services are put into action. Countries may successfully enhance maternal health outcomes and contribute to the well-being of mothers and children throughout their communities by identifying and addressing the socioeconomic determinants of maternal health.

### What is known about this topic

- *Maternal health outcomes are influenced by social determinants, and understanding these factors is crucial for improving maternal health globally;*
- *Social determinants of maternal health exhibit significant variability across geographical regions and within individual countries;*
- *The Middle East and North Africa (MENA) region experiences diverse social determinants impacting maternal health outcomes*.

### What this study adds

- *A comprehensive scoping review identified 50 articles specifically analyzing social factors affecting maternal health outcomes in the Middle East and North Africa region;*
- *The study highlights a range of social factors, including conflict zones, rural residence, healthcare accessibility and quality, education levels, familial relationships, cultural practices, household wealth, financial security, childbirth history, and interpersonal violence, that independently or collectively influence maternal health outcomes in the Middle East and North Africa region;*
- *The study emphasizes the need for multisector collaboration involving governmental ministries, non-governmental organizations, local authorities, healthcare delivery programs, and community members to address traditional practices, enhance healthcare accessibility and affordability, and create sustainable solutions for maternal health in Middle East and North Africa nations*.
